# Clinician Experiences with Adolescents with Comorbid Chronic Pain and Eating Disorders

**DOI:** 10.3390/jcm14155300

**Published:** 2025-07-27

**Authors:** Emily A. Beckmann, Claire M. Aarnio-Peterson, Kendra J. Homan, Cathleen Odar Stough, Kristen E. Jastrowski Mano

**Affiliations:** 1 Department of Psychology, College of Arts & Sciences, University of Cincinnati, Cincinnati, OH 45221, USA; ebeckmann@brownhealth.org (E.A.B.); odarcc@ucmail.uc.edu (C.O.S.); 2 Department of Psychiatry & Human Behavior, Warren Alpert Medical School of Brown University, Providence, RI 02903, USA; 3 Cincinnati Children’s Hospital Medical Center, Cincinnati, OH 45229, USA; claire.aarnio-peterson@cchmc.org (C.M.A.-P.); kendra.homan@cchmc.org (K.J.H.)

**Keywords:** chronic pain, eating disorders, pediatric, assessment, treatment

## Abstract

**Background/Objectives**: Chronic pain and eating disorders are two prevalent and disabling pediatric health concerns, with serious, life-threatening consequences. These conditions can co-occur, yet little is known about best practices addressing comorbid pain and eating disorders. Delayed intervention for eating disorders may have grave implications, as eating disorders have one of the highest mortality rates among psychological disorders. Moreover, chronic pain not only persists but worsens into adulthood when left untreated. This study aimed to understand pediatric clinicians’ experiences with adolescents with chronic pain and eating disorders. **Methods**: Semi-structured interviews were conducted with hospital-based physicians (*N* = 10; 70% female; *M* years of experience = 15.3) and psychologists (*N* = 10; 80% female; *M* years of experience = 10.2) specializing in anesthesiology/pain, adolescent medicine/eating disorders, and gastroenterology across the United States. Audio transcripts were coded, and thematic analysis was used to identify key themes. **Results**: Clinicians described frequently encountering adolescents with chronic pain and eating disorders. Clinicians described low confidence in diagnosing comorbid eating disorders and chronic pain, which they attributed to lack of screening tools and limited training. Clinicians collaborated with and consulted clinicians who encountered adolescents with chronic pain and/or eating disorders. **Conclusions**: Results reflect clinicians’ desire for additional resources, training, and collaboration to address the needs of this population. Targets for future research efforts in comorbid pain and eating disorders were highlighted. Specifically, results support the development of screening tools, program development to improve training in complex medical and psychiatric presentations, and methods to facilitate more collaboration and consultation across health care settings, disciplines, and specialties.

## 1. Introduction

Chronic pain and eating disorders are two highly prevalent conditions affecting adolescents. According to national estimates, 6% to 26% of children and adolescents between the ages of 6 and 17 experience chronic pain [1,2]. Eating disorders (EDs) are diagnosed in approximately 3.8% of adolescent females and 1.5% of males in the United States [3]. Moreover, approximately 13% of adolescents will develop an eating disorder upon transitioning to young adulthood [4]. Chronic pain, especially chronic functional abdominal pain, might put an individual at risk for the development of an eating disorder. A variety of pain-related symptoms, such as reduced appetite, dietary changes, nausea, and avoidance of movement can impact nutrition and weight, which in turn could trigger the development of an eating disorder [5,6]. Not only has it been demonstrated that adolescents with chronic pain are at risk for eating disorders, but the reverse pattern has also been noted. Specifically, adolescents with eating disorders can develop chronic pain [7]. Gastrointestinal distress is common among children and adolescents with eating disorders [7,8,9,10]. Research cites that as much as 25% of children and adolescents with restrictive eating also experience ongoing gastrointestinal distress, discomfort, and pain [11].

There are many shared risk factors that likely contribute to the co-occurrence of chronic pain and eating disorders in adolescents. It is possible that as a result of shared risk factors, adolescents with chronic pain are at increased risk for developing restrictive eating, and vice versa. Individuals with chronic pain and eating disorders possess shared temperamental risk factors, such as tendencies toward perfectionism [12] and harm avoidance [13]. Social factors also increase the likelihood of developing eating disorder pathology in adolescents with chronic pain [11,12]. Social reinforcement of weight loss is linked to increased frequency of restrictive eating behaviors to maintain low weight [6].

Despite the association between chronic pain and eating disorders, the identification, assessment, diagnosis, and treatment of comorbid chronic pain and eating disorders is challenging for clinicians. Sim and colleagues [6] found a significant delay in identification of eating disorders in adolescents with chronic pain compared to adolescents with an eating disorder without chronic pain. Hampered diagnosis is particularly problematic, as early detection is critical in both pediatric chronic pain [14] and eating disorder treatment effectiveness [15]. Postponed treatment has been linked to increased likelihood of pain and eating disorder symptoms becoming persistent, higher health care costs, and lower quality of life [15,16,17]. Moreover, eating disorders, particularly anorexia nervosa, have one of the highest mortality rates of any psychiatric disorder [18,19]. These statistics become incredibly salient in the context of comorbid eating disorders and chronic pain, as these individuals are diagnosed significantly later than those without the comorbidity [6].

Although no research has examined methods for identifying and assessing for co-occurring chronic pain and EDs, it is common practice for clinicians to conduct a physical exam and psychological assessment for patients with chronic pain or eating difficulties [20]. Comorbid chronic pain and eating disorders are likely difficult to diagnose, as many physical symptoms overlap [6]. Both conditions can involve weight fluctuation, food refusal, fasting, fatigue, orthostatic intolerance, early satiety, reflux, and/or constipation [21]. Despite such similarities, certain symptoms could serve as differentiators between the two conditions.

Sim and colleagues [6] highlighted behavioral signs of eating disorders for medical professionals to consider when seeing a patient with chronic pain who may also have an eating disorder. Behaviors such as tracking calories, having a calorie “goal” without input from a medical professional, excessive exercise, and body checking are often present in the context of an eating disorder, but are unlikely to be present in adolescents with chronic pain alone. Similarly, an intense fear of weight gain and significant body dissatisfaction are often reported by adolescents with anorexia nervosa [21] but would not likely be experienced by adolescents who have chronic pain without anorexia nervosa. A different eating disorder diagnosis also characterized by restrictive eating is avoidant restrictive food intake disorder (ARFID), which does not require body dissatisfaction or desire for weight loss for diagnosis. Indeed, identifying comorbid chronic pain and eating disorders is not a straightforward process. Despite the challenges associated with this complex presentation, no studies have explored clinician experiences in identifying, assessing, and treating comorbid chronic pain and eating disorders. Given the prevalence of eating disorders and chronic pain among adolescents, it is imperative to understand clinician experiences across specialties and care settings to improve the efficiency, accuracy, and ease with which clinicians assess, diagnose, and treat adolescents with comorbid chronic pain and eating disorders. Quickly addressing these concurrent, dangerous, and complex conditions could prevent mortality as well as unnecessary health care costs and low quality of life.

### Present Study

Despite knowledge of the co-occurrence of chronic pain and eating disorders [11,22] and the danger of delayed diagnosis [6], there remains a lack of research on factors impacting identification, assessment, and treatment of comorbid chronic pain and eating disorders. Moreover, chronic pain and eating disorder patients present to several different pediatric clinics, yet no studies have considered cross-discipline clinician experiences with this population. Improved understanding of clinician perspectives on their work with adolescents with chronic pain and eating disorder symptoms is imperative for the rapid identification of symptoms, prevention of disease progression, and the advancement of treatment approaches. Additionally, determining clinician-identified strengths and weaknesses in professional education, training, and collaborative experiences could inform program development and refinement to support providers in building competence and confidence with this population.

The aim of the current study was to describe cross-disciplinary clinician experiences with adolescents who present with chronic pain and eating disorder symptoms in interdisciplinary academic medical center settings across the United States. The current study employed a cross-sectional phenomenological qualitative study design and utilized multi-disciplinary sources of reporting.

## 2. Materials and Methods

### 2.1. Study Questions, Design, and Setting

Consistent with phenomenological design, questions informed by emerging research were posed instead of hypotheses so that exploration of the phenomenon was not limited [23]. The exemplar study involved three primary research questions: (1) What are clinician experiences with adolescents with chronic pain and eating disorders? (2) What educational and training experiences in chronic pain and eating disorders have clinicians obtained throughout their careers? and (3) What experiences do clinicians have in consultation and collaboration with other providers when working with adolescents with chronic pain and eating disorders?

### 2.2. Participants

Participants were hospital-based clinicians at several academic medical centers across the United States. Participants were practicing physicians and psychologists with experience in identification, assessment, diagnosis, and/or treatment of adolescents with comorbid chronic pain and eating disorders. Inclusion criteria for the current study included the following: (1) participants were practicing physicians or psychologists in an academic medical center in the United States at the time of participation, (2) participants were required to have previous clinical experience with adolescents with chronic pain and eating disorders (the population of interest for the interview), (3) participants were English-speaking (language of the interview), (4) participants had required access to a device with video conferencing capabilities (participation took place over Zoom).

### 2.3. Participant Sampling and Recruitment

Participants were recruited using convenience, purposive, and snowball sampling techniques. Convenience sampling was the first techniques used to identify potential study participants in the selected pediatric academic medical centers. Pediatric academic medical centers were selected based on the presence of specialty clinics for eating disorders, gastroenterology, and/or pediatric pain as well as the presence of physicians and psychologists in the clinics. Study flyers were distributed to division directors in adolescent medicine, gastroenterology, and anesthesiology at several pediatric academic medical centers across the United States. Directors distributed flyers to physicians and psychologists working in the divisions and specialty clinics.

Purposive sampling and snowball sampling techniques were used to recruit participants following the distribution of study flyers. Purposive sampling allows researchers to identify and select participants who have direct experience with a phenomenon of interest, in this case, adolescents with eating disorders and chronic pain [24,25]. Snowball sampling allows for participants to recommend other contacts who might be interested in participating in the study using their own networks [26]. Interested clinicians were encouraged to contact the principal investigator briefly describing their experience with the population of interest. Consistent with previous research studies utilizing interview techniques within a phenomenological approach and a narrow scope, a sample size of 20 clinicians-physicians and psychologists was decided upon for the current study [27].

Prior to participation, the research team informed each participating clinician via email that they would be asked questions about their clinical experiences with adolescents with chronic pain and eating disorders. It was also explained that the interview audio would be recorded for transcription purposes and that the interview recordings would be de-identified and stored on a private server.

### 2.4. Semi-Structured Interview Guide

A semi-structured interview guide (see Table 1) was formulated by the research team based on the research questions. The guide was field tested with a physician and a psychologist with clinical experience with adolescents with chronic pain and eating disorders. The physician and psychologist completed field testing independently so as not to influence one another’s feedback. Feedback supported clarity and comprehensibility of the semi-structured interview guide. No amendments or modifications were made following field testing.

### 2.5. Interview and Data Acquisition

Interviews were open-ended, semi-structured interviews and conducted one-on-one with the PI and each participant. Interviews took place over the course of a 3-month period (January 2023–March 2023). The semi-structured interview guide allowed for the exploration of specific topics using pre-written interview questions, while also allowing for flexibility throughout the interview process to explore connected themes and spontaneous follow-up questions [28]. Interviews took place for approximately one hour with each clinician and the PI. The Zoom interview method was used for ease of recording and to afford each clinician the opportunity to participate at a time most convenient for them. In addition to the interview data, the PI gathered demographic information (e.g., gender identity, number of years of experience). Participants were compensated for their time with a $25 Amazon gift card.

### 2.6. Data Analysis

All audio recordings were transcribed verbatim using NVivo transcription software (V.13). Three researchers were involved in the data analytic process, including the PI and two undergraduate research assistants trained in qualitative data analysis and coding techniques. Consistent with the Braun and Clarke [29] approach to thematic content analysis, analysis was conducted in four phases. The four phases of thematic content analysis included the following: (1) reviewing of transcripts for errors and saturation, (2) initial coding, (3) code grouping and theme identification, (4) reviewing of themes [29].

**Figure 1:**
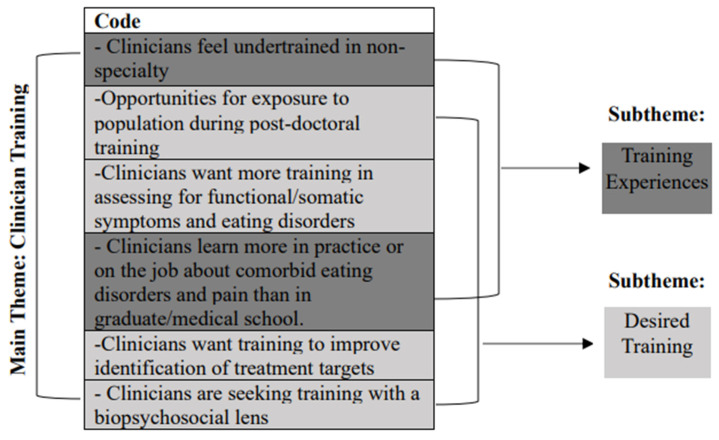
Example of grouped code assignment to main themes and subthemes.

The first phase involved reviewing the auto-transcription of de-identified interviews to correct any errors in transcription (e.g., “are feed” instead of ARFID). This step also involved the reading and re-reading of transcripts to evaluate thematic saturation and data quality. Researchers highlighted quotes of significance and key terms related to the research questions. Phase two involved initial coding using a hybrid method of deductive and inductive coding approaches. Each member of the research team independently coded each transcript to allow for the evaluation of intercoder reliability. The research team also met together at three separate times to assess intercoder agreement. Phase three focused on code grouping and theme identification. The research team collaboratively sorted through codes and grouped overlapping codes. Codes were grouped if a recurring pattern was observed across transcript data around a particular topic. The PI independently assigned the grouped overlapping codes into main themes and subthemes (see Figure 1 for an example). All identified themes were organized into a table with relevant codes and participant quotes (see Appendix A). The goal of phase four was to review identified main themes and subthemes. The research team collaboratively reviewed each main theme and subtheme in the table for agreement and finalization.

## 3. Results

### 3.1. Participants

Demographic information and participant characteristics for the study sample are summarized in Table 2. The final sample consisted of physicians (*n* = 10; *M* years of experience = 15.3) and psychologists (*n* = 10; *M* years of experience = 10.2) at several academic medical centers across the United States.

### 3.2. Themes

#### 3.2.1. Main Theme 1: Clinical Practice

Codes falling under the main theme of clinical practice included statements from clinicians regarding where and how patients and their families presented for screening, assessment, and treatment. Moreover, clinicians described approaches to screening, assessment, and treatment and proposed approaches that might be effective to address comorbid chronic pain and eating disorders in the future.

##### Subtheme 1: Patient Presentation

Clinicians stated that the most common pain condition among patients they encounter when they see adolescents with comorbid chronic pain and eating disorders is chronic functional abdominal pain (CFAP). The majority of participating clinicians also noted that the most common eating disorder experienced by patients with comorbid chronic pain and eating disorders is avoidant restrictive food intake disorder (ARFID). Clinicians also spoke to the types of symptoms endorsed by patients when they presented to clinic. Specifically, clinicians shared that patients who sought care for chronic pain also endorsed eating disorder symptoms. Alternatively, some patients who sought care for an eating disorder were also found to have chronic pain symptoms. Clinicians commented on caregiver reports and perceptions at the time of patient presentation. Moreover, clinicians noted that caregivers often expressed fatigue and frustration related to their child’s symptoms. Caregivers also disclosed feelings of guilt and fear to clinicians in the context of treatment efforts and symptom management.

*“By the time these patients get to me, the ones with functional pain, they’ve had the ultrasounds, the scopes, the colonoscopies… and they still don’t have answers as to what’s causing the pain. Then you have to tell them that there might not be an organic cause. That’s really tough.”*

(Physician, Anesthesiology)

##### Subtheme 2: Screening and Assessment

The subtheme of screening and assessment captures clinician experiences in the evaluation of adolescents with comorbid chronic pain and eating disorders. Codes categorized under this subtheme also highlight clinicians’ thoughts on what might be helpful in terms of improving screening and assessment of adolescents with chronic pain and eating disorders. Clinicians emphasized the importance of asking questions beyond the diagnosis(es) in their medical chart, especially if there are suspicions of a comorbid disorder. No participants reported the use of formal measures during the screening and assessment of adolescents with chronic pain and eating disorders. Despite the lack of validated screening and assessment measures, the majority of clinicians emphasized the importance of taking a careful and thorough history of symptoms.

*“I’m looking at the behavior. If the kid is saying pain is impacting eating and daily activity yet is exercising a ton and eating foods that tend to cause gas and bloating, like cauliflower rice and sugar free stuff, I’m paying attention… alarm bells are going off.”*

(Psychologist, Pain)

Other notable topics addressed by clinicians regarding screening and assessment included determining what is driving food avoidance, challenges associated with teasing apart symptoms of pain and eating disorders, as well as the need for caregiver involvement in the screening and assessment process.

##### Subtheme 3: Intervention

Clinicians spoke to relevant treatment targets and factors impacting treatment in comorbid chronic pain and eating disorders. Transdiagnostic approaches were also proposed. Several clinicians spoke to challenges related to effective intervention in adolescents with chronic pain and eating disorders. Patients’ ability to participate in treatment was discussed by clinicians with an emphasis on medical stability. For example, if a patient is severely malnourished, they might not be able to engage in the physical demands associated with pain rehabilitation. The impact of caregiver buy-in to patient diagnosis(es) on intervention was also addressed. Clinicians proposed Family Based Treatment (FBT), a combination of Acceptance and Commitment Therapy and Cognitive Behavioral Therapy (CBT), as well as exposure therapy as potential intervention approaches to address both chronic pain and eating disorders. Lastly, clinicians spoke to the use of tube feeding and medications as other treatment options for patients.

*“FBT is the gold standard as you know for eating disorder treatment. I still think it’s relevant for these kids who also have pain issues. These kids need to be eating and having parents take control is the best way to ensure that, because if we leave it up to the eating disorder or pain the kid isn’t going to eat.”*

(Physician, Adolescent Medicine)

#### 3.2.2. Main Theme 2: Clinician Training

Clinician training represents clinician training experiences in graduate school, medical school, residency, and post-doctoral training, as well as peri-career training. The theme of clinician training also reflects desired training opportunities expressed by clinicians.

##### Subtheme 1: Training Experiences

In terms of training experiences, clinicians expressed feeling undertrained in their non-specialty. Physicians specifically noted that they felt undertrained in functional pain disorders and somatic symptoms. Psychologists expressed minimal training in graduate school for eating disorders. Additionally, clinicians shared that they learned more in practice and in their work than they learned in medical school or graduate school on complex presentations of chronic pain or eating disorders. Clinicians described previous discussions with colleagues about non-specialty cases, shadowing other clinicians at work. Many clinicians sought consultation and supervision to train in non-specialty areas.

*“I have career long experience with eating disorders. In regard to pain, I don’t have any training. Of course, I have attended some workshops. I’ve talked with colleagues at the hospital.”*

(Physician, Adolescent Medicine)

##### Subtheme 2: Desired Training

Many clinicians spoke to training experiences that they desired or wished that they could have received earlier than post-doctoral training. Specifically, opportunities for exposure to their non-specialty in medical school and graduate school were endorsed. Clinicians shared that they wanted more training to assess for functional/somatic symptoms and eating disorders. Additional training experiences sought by clinicians included training to improve treatment targets and training with a biopsychosocial lens.

*“It’s unfortunate, because the pain these kids have is real and we don’t always do a great job of validating functional stuff in the medical world. I’m not confident that I have the best language to respond when kids come in with functional pain or somatic symptoms. I would love more training on that.”*

(Physician, Adolescent Medicine)

#### 3.2.3. Main Theme 3: Collaboration and Consultation

The main theme of collaboration and consultation entails clinician comments addressing referring patients to different specialty clinics and providers, the setting(s) in which they see patients, and collaboration with other providers.

##### Subtheme 1: Referrals

Clinicians reported referring cases to other providers and clinics when they did not feel like they had the expertise to diagnose and treat the patient. Clinicians described awareness of their competency. They also shared their preference to refer patients out when they felt that they might be practicing out of their scope.

*“If ever I felt like someone needed true treatment for an eating disorder, I’m going to refer because I’m not, you know, I don’t have the expertise for that. So I don’t typically do the weight focus. I just say we have to stop the weight loss. How do we do that? Let’s come up with some strategies and go from there.”*

(Psychologist, Gastroenterology)

##### Subtheme 2: Care Setting

Communication with other providers in different care settings was addressed by participating clinicians. Physicians discussed communication with mental health professionals in the community and within the academic medical center setting. Psychologists also discussed communicating with community providers on complex cases where the patient was also seen at an academic medical center. Moreover, clinicians highlighted the strengths associated with working with adolescents with comorbid chronic pain and eating disorders in an interdisciplinary setting. Clinicians expressed the difficulty in finding a higher level of care options for adolescents with comorbid chronic pain and eating disorders, as many inpatient pain rehabilitation programs do not accept adolescents with eating disorders, and inpatient eating disorder programs often do not accept adolescents with a co-occurring chronic pain diagnosis.

*“In the inpatient intensive rehab program for kids who have been affected by chronic pain, you can’t walk, is in a wheelchair, can’t attend school, they manage everything except for eating. You must be able to eat. Otherwise, they’re not able to participate, which makes no sense to me. They have to be strong enough nutritionally to do physical therapy, but they can’t restore nutritionally because of chronic pain. Our eating disorder program doesn’t accept kids with chronic pain either.”*

(Physician, Adolescent Medicine)

##### Subtheme 3: Collaboration

Collaboration was addressed by participating clinicians. They described feeling confident in knowing who to ask or consult within an interdisciplinary team if they felt uncertain or wanted advice on a patient with comorbid chronic pain and eating disorders. They also spoke extensively about defining roles within the treatment team. Clinicians collaborated with the patient’s treatment team and saw the benefit in allowing each team member to address patients’ needs in their domain. For example, psychologists described referring to adolescent medicine for questions related to medical stability and to dietitians for caregiver questions about calorie requirements and meal plans.

*“It’s so important to collaborate on our teams. I don’t know how many calories a patient should be consuming. I don’t have that expertise, so I loop in their dietitian if there are questions. I also don’t know if they’re orthostatic by heart rate or blood pressure unless they’re following with a pediatrician. We need to work together.”*

(Psychologist, Eating Disorders)

## 4. Discussion

The present study was the first to explore physician and psychologist experiences working with adolescents with comorbid chronic pain and eating disorders. A qualitative design was used with deductive and inductive approaches to thematic analysis to characterize cross-disciplinary clinician experiences with this population. Results support existing research suggesting that patients can develop pain symptoms prior to and after the onset of eating disorder symptoms [6]. Findings not only demonstrate how patients present with comorbid chronic pain and eating disorders, but also how their caregivers present. Specifically, clinicians reported awareness of caregiver emotions and wellbeing. Per clinician report, caregivers presented with treatment fatigue and frustration, as well as fear and guilt. Research has demonstrated a relationship between caregiver expressed emotion and treatment outcomes in adolescents [30,31]. Taken together, these findings emphasize the salience of family-centered care for adolescents with comorbid chronic pain and eating disorders.

Clinicians noted their desire for screening tools [32] and described their rationale for examining behavioral signs of pain and eating disorders as a critical component of the assessment process. This is consistent with guidelines provided by Sim and colleagues for individuals working with adolescents with chronic pain and eating disorders [6], which encouraged the use of screening tools in the assessment process and included a list of behavioral signs of eating disorders. Clinicians who participated in the present study also commented on the challenges associated with teasing apart symptoms of pain and eating disorders. Consistent with anticipated diagnostic features, clinicians spoke to weight loss, avoidance, functional disability, food restriction, constipation, and body dissatisfaction. Moreover, clinicians did not endorse using specific assessment approaches.

Regarding intervention, clinicians emphasized the effectiveness and utility associated with FBT, CBT, and exposure therapy for comorbid chronic pain and eating disorders. Previous research demonstrated the use of these interventions for pediatric chronic pain, with the exception of FBT [33,34]. Evidence has also been shown for the effectiveness of CBT and exposure therapy for the treatment of eating disorders [35,36,37], but research has not examined the effectiveness of these treatment modalities for co-occurring pain and eating disorders. Many clinicians hypothesized that adapted versions of these interventions could be effective at treating chronic pain and eating disorders simultaneously if the adolescent is medically stable.

Clinicians described feeling undertrained in their non-specialty. For example, physicians and psychologists who worked in adolescent medicine and eating disorders noted that they did not receive much training in functional pain or somatic symptom disorder during medical or graduate school. Indeed, existing research supports that clinicians do not receive significant graduate level training in somatic symptoms [38,39]. The present study builds upon this literature, as the majority of clinicians desired more training in complex presentations of functional pain and eating disorders at the medical school and graduate level.

Clinicians reported a preference to refer cases to other providers and clinics when they did not feel like they had the expertise to diagnose and treat the patient. Regarding the care setting subtheme, communication with other providers in different care settings was addressed by participating clinicians. Physicians and psychologists shared that they communicate with community providers and within the academic medical center setting on complex cases. An important finding related to the referral and care setting subtheme is that low perceived competence and confidence in working with patients with complex presentations increased the likelihood of clinicians referring patients to clinicians in other disciplines and specialties. Clinicians also shared that they felt confident in knowing who to ask or to consult within an interdisciplinary team if they felt uncertain or wanted advice on a patient with comorbid chronic pain and eating disorders. Together, these findings suggest that collaboration was helpful to increase clinician confidence. However, training is what clinicians linked to increasing their competence in addressing adolescents with this comorbid chronic pain and eating disorders.

### 4.1. Clinical Implications

Findings from the present study demonstrate the complexity of patient presentation as well as variability in approaches to screening, assessment, diagnosis, and treatment of adolescents with comorbid chronic pain and eating disorders. Clinicians could benefit from increased development and use of practical and simple screening and assessment tools, such as the recently validated Eating Attitudes Test–16–Chronic Pain [32]. Moreover, results highlight the impact that previous health care (e.g., medical testing, treatment fatigue, medicalization of patient) can have on patient and family presentation.

Results also highlight the lack of training at the medical school and graduate level and that training at this level is desired by clinicians. Future program development efforts might aim to build in education for functional pain and somatic symptoms as well as diversity in eating disorder presentations for physicians and psychologists at the graduate level. Additionally, internship/residency and post-doctoral programs could increase training opportunities on rotations to increase exposure and consequently confidence in working with complex presentations of medical and psychiatric conditions.

Several themes captured the importance of an interdisciplinary setting for the care of adolescents with comorbid chronic pain and eating disorders. Research should determine barriers to the development of outpatient, partial hospitalization, and inpatient interdisciplinary pain and eating disorder programs in order to provide adolescents with comprehensive care at a level that is most appropriate for the acuity of their condition and to meet the needs of the patient and their family.

### 4.2. Limitations and Future Directions

It is imperative to understand the limitations of the present study. Although we reached thematic saturation with 20 participants, the sample of physicians and psychologists was small. Thus, we were unable to examine, for example, differences in responses based on when clinicians completed their medical and subspecialty training. Further, all anesthesiologists interviewed in our study practiced within integrated pediatric pain clinics that were structured around interdisciplinary models of care, involving collaboration among physicians, psychologists, and other specialists. Though this recruitment approach was deliberate given that interdisciplinary approaches are essential when treating youth with complex conditions, the perspectives of clinicians working in family medicine, community clinics, and private practice may differ from those of clinicians working with adolescents with chronic pain (and/or eating disorders) in interdisciplinary clinics in academic medical centers. Future research might also aim to obtain a wider sample of providers, including pain medicine physicians, psychiatrists, nurses, physiotherapists, and dietitians.

An additional limitation related to the study design is that the interviews were conducted virtually. Although the Zoom method increases accessibility, as participants do not have to travel to a research lab, virtual interviews could impact participant comfort and also require that participants have specific technology [40]. Participants were required to have access to the internet, a device with video conferencing capability, and a secure space. Therefore, consideration of the impact on available office space and/or devices for potential participants was important. Also, important to note, all interviews took place with an interviewer who is in the psychology field, which could have impacted physician willingness to share negative experiences associated with psychology colleagues. Future studies may consider matching interviewer and participant on this domain, or perhaps conduct group interviews or focus groups with participants representing varied training backgrounds and areas of expertise.

## 5. Conclusions

The current study contributes to the existing literature by providing insight into clinicians’ experiences with adolescents with comorbid chronic pain and eating disorders. Results also indicate clinicians’ desire for more graduate and post-doctoral training in somatic symptoms, functional pain, and eating disorders. Participants emphasized the importance of working collaboratively with other specialties and disciplines to increase clinician confidence and competence in the identification, assessment, and treatment of adolescents with chronic pain and eating disorders.

Results highlight future directions for the development of specific screening questions, assessment tools, and intervention strategies for adolescents with comorbid chronic pain and eating disorders. Moreover, this study presents targets for improved education and training in pediatric functional pain and eating disorders as well as opportunities to optimize collaboration and consultation across medical and psychiatric settings, disciplines, and specialties.

## Figures and Tables

**Table 1:** Structured Interview Guide.

Focus Area	Questions	Probes
**Clinical Experiences**	- Describe your experiences with patients who have chronic pain and eating disorder symptoms.	- Can you tell me more about it? - Do you have any other experiences that you’d like to share?
	- Recount a specific experience where you encountered a patient with chronic pain and eating disorder symptoms.	- Describe the methods you used to identify the symptoms. - How did you go about making their diagnosis/es? - Describe your recommendations for treatment.
**Training**	- Discuss your training in working with the chronic pain and eating disorder populations.	- Do you feel like you received an adequate amount of training in the two disciplines? - Describe the strengths of your training. What did you like about it? - Describe any weaknesses in your training. What do you wish you could have changed (if anything)? - Describe your confidence as it relates to identifying and diagnosing chronic pain and eating disorders upon completing your training.
**Collaboration**	- Express your thoughts and feelings regarding an interdisciplinary approach to diagnosing and treating patients with chronic pain and eating disorder symptoms.	- Do you often consult with other clinicians or professionals prior to making a diagnosis? - If so, what benefits or drawbacks have you noticed from working as a part of an interdisciplinary team? - If not, do any barriers or limiting factors exist to consulting with other professionals and clinicians?

**Table 2:** Sample Characteristics (*N* = 20).

Variable	*N* (%)
**Sex**	
Male	13 (65)
Female	7 (35)
**Race**	
White	10 (50)
Asian	3 (15)
Black	2 (10)
Latine	1 (5)
More than one race	1 (5)
**Location of Academic Medical Center**	
Midwest	9 (45)
West	5 (25)
Northeast	4 (20)
South	2 (10)
**Specialty**	
*Physician*	
Adolescent Medicine	4 (40)
Anesthesiology	4 (40)
Gastroenterology	2 (20)
*Psychologist*	
Pain	6 (60)
Eating Disorders	3 (30)
Gastroenterology	1 (10)

## Data Availability

The data underlying this article cannot be shared publicly due to the privacy of the individuals who participated in the study. The data will be shared by the corresponding author on reasonable request.

## Abbreviations

The following abbreviations are used in this manuscript:

ED	Eating Disorder
AN	Anorexia Nervosa
FBT	Family based treatment
CBT	Cognitive behavioral therapy

## Appendix A

**Table A1:** Theme 1: Clinical Practice Themes, Codes, and Quotes.

Subtheme	Code(s)	Physician Quotes	Psychologist Quotes
Patient Presentation	CFAP and ARFID are the most common presentations.	(ADOLMED_2) The most common way I see a patient who has both an eating disturbance and chronic pain is when they’re referred to me by GI to rule out ARFID, which is one of the most common eating disorders from people who have gastrointestinal problems.	(PAIN_4) They [patients] present for chronic pain problems and during the assessment or in reviewing the medical record there is evidence of weight loss due to chronic functional abdominal pain and food avoidance, because of loss of appetite or nausea or something like that. That’s usually where I first encounter them.
Patient Presentation	Some patients present first with pain symptoms and then develop ED symptoms.	(ADOLMED_4) The adolescent brain is very vulnerable, so sometimes just the fact that they lost weight from avoiding food or pain could trigger their eating disorder. (ANESTH_6) I see kids come in with abdominal pain usually a secondary pain complaint to something like headaches or MSK pain… those kids are the ones who would fit this category.	(PAIN_7) The kids I see, see me for help with their pain and then I come to find that they’ve lost a bunch of weight and are avoiding food trying to avoid pain… and sometimes the weight loss is praised by peers, which can be reinforcing to the food restriction.
Patient Presentation	Some patients present first with ED symptoms and then develop pain symptoms.	(ADOLMED_7) In the process of losing weight, then they had secondary GI issues that cause the constipation, the gastroparesis, the delayed gastric emptying, whatever we call it, the stomach pain as you start to refeed. (GI_10) I get a mix of kids who have an eating disorder diagnosis already and are worried about a secondary GI issue. I also get kids with GI pain who also have some restrictive behaviors and more eating stuff.	(EATING_6) Parents come in with their child who is refusing to try new foods and is afraid of foods for textural reasons or something like that. Then we find that they’re complaining about stomach pain. (EATING_3) It’s definitely a chicken or the egg kind of situation, like obviously during the refeeding process, there’s going to be GI discomfort and pain.
Patient Presentation	Patients/families present with treatment fatigue and frustration	(ANESTH_5) By the time these patients get to me, the ones with functional pain, they’ve had the ultrasounds, the scopes, the colonoscopies… and they still don’t have answers as to what’s causing the pain. Then you have to tell them that there might not be an organic cause. That’s really tough.	(EATING_8) So many parents are frustrated, their child is losing weight and they’ve tried making them eat and they’ve tried accommodating their preferences or pushing back on them... they’re often scared and exhausted once they get to us.
Patient Presentation	Parents feel guilt and fear	(ADOLMED_3) Parents often express fears like, “Can I do this? I’m afraid they’ll refuse food.” They also express guilt, like they say things like, “I wasn’t paying enough attention to my kid. I should have brought them in earlier.” (ANESTH_5) It’s tough, because parents feel like it’s mean to make them [their child] do something that causes pain.	(EATING_3) Many parents say things like, “I can’t feed my kid… they’re malnourished, they keep losing weight… I should have noticed earlier.” (PAIN_9) Parents feel awfully guilty when they realize that they’ve unintentionally enabled their child’s avoidance. One example is if they let their kid stay home from school day after day because of pain.
Patient Presentation	Patients rarely present with chronic pain or EDs alone. Often comorbidities.	(GI_10) We do sometimes have someone with an eating disorder and chronic pain, and we have a lot of kids with a specific type of eating disorder called ARFID. Many of them report chronic abdominal pain. Fewer of them have chronic pain in general, but a lot of them have like chronic abdominal pain and anxiety or abdominal pain and functional nausea.	(PAIN_9) Very few kids are engaging only in eating disorder behaviors or only have chronic pain. Most of the time, there’s something else that triggered it or came along with it, like anxiety for example. Some kids also have depression, OCD, and/or other somatic complaints.
Screening and Assessment	Important to ask questions beyond primary diagnosis, especially if suspected comorbidity.	(ANESTH_1)…A lot of times the kids come pre-labeled. Every once in a while, we’ll get a kid where you will ask some generic stuff and they’ll go, “Well, I’ve lost some weight.” We’ll [physicians] look at the growth curve, really quick and go, yeah, you know, you’ve lost 10 pounds in the last six months. Did you do this on purpose or have you been otherwise unwell?	(EATING_8) Sometimes kids come in with diagnoses and it’s not that I’m questioning those… I just think it’s important to keep an open mind. We need to ask all of the questions, because they often meet criteria for an additional diagnosis that would involve different interventions.
Screening and Assessment	No formal measures are used in screening and assessment.	(ADOLMED_4) I don’t use any questionnaires or measures to assess for an underlying pain condition. I just ask follow-up questions if they describe any pain. For example, I might ask how often they’re stooling. If they’re experiencing pain related to constipation, I’ll recommend an osmotic laxative.	(PAIN_4) I have no formal measures that I’m administering, and it probably falls along the same lines as if abdominal pain is the primary presenting problem. I definitely do the eating assessment as part of their new visit in pain clinics or if I flag the chart from a GI, if I flag like a weight loss issue, that even if abdominal pain is not the primary pain complaint, if they have widespread pain, but they’ve seen GI and there is a weight loss issue, then I do more of the assessment. (PAIN_2) Truthfully, like, we don’t have screening measures in that clinic and that’s a problem. In the context for that patient and for similar patients, what I really learned to do was to look at the growth chart and see change in weight status over the course of time. And if I could see that there was a drop that would immediately raise my red flags, I would immediately be concerned.
Screening and Assessment	Clinicians acquire history of symptom presentation.	(GI_8) I like a sense of the history of onset and change of behaviors to cause, I think sometimes that’s helpful if there... was it the pain first or was it that you were restricting and then you started to notice more pain or noticed more pain and if it’s chronic it might have been there like long standing,	(EATING_3) It’s really important for me to think about when things started to come up for them… did the pain symptoms come up before food restriction or was it the food restriction and eating issues that preceded the pain symptoms? It helps me to better conceptualize the presenting problem. (GI_5) I think it’s very hard, because these symptoms bleed into one another… that’s why I want to ask questions to get at what came first. I guess it’s possible that things develop at the same time. These are things I’m trying to figure out.
Screening and Assessment	Even if a patient does not endorse ED symptoms, behaviors might indicate the presence of pathology.	(GI_10) Looking at the behavior is really helpful with teens in particular, because I think sometimes they not only lack insight, but then also it’s like sometimes they’re not going to be the most forthcoming. For example, they don’t always say “I would like to be a lot skinnier” or “I have body image issues”, but they might be willing to share, oh, “I don’t want to eat snacks like that.” (ADOLMED_2) If someone wants to lie, it doesn’t matter what I ask them. their behaviors can often tell us a lot. They can sleep through the night and get continuous feeds without pain, but during the day, if you try to feed them through an NG tube and every time you come into the room, the pump has been shut off because they say they “couldn’t take the pain anymore”. That’s someone I would have a higher oh, you probably have an eating disorder.	(PAIN_10) When I bring up eating and doing an intervention, if I don’t get a lot of resistance, I’m not thinking about the eating piece because I’m not getting 20 reasons why it won’t work. It’s not systematic. I’m not doing a body image assessment or using any set questionnaires that might assist me. It’s kind of like how things unfold during treatment. (PAIN_7) I’m looking at the behavior. If the kid is saying pain is impacting eating and daily activity yet is exercising a ton and eating foods that tend to cause gas and bloating, like cauliflower rice and sugar free stuff, I’m paying attention… alarm bells are going off.
Screening and Assessment	Determine what is driving food avoidance.	(ADOLMED_7) I try to figure out what the function of like avoiding eating would be and some of the behaviors. So asking like, OK, so you are skipping meals tell me a little bit about like what that was like and why. I think being able to kind of figure out what’s prompting the behaviors and the reason is sometimes helpful because there have been times where patients have just been like, “I don’t want to be in pain.” That changes my conceptualization.	(GI_5) I try to figure out what the function of like avoiding eating would be and some of the behaviors. I’m so asking like, OK, so you were kind of like skipping meals like tell me a little bit about like what that was like and why. And I’ve been able to kind of figure out what’s prompting the behaviors and the reason is sometimes helpful because there have been times where patients have just been like, I don’t want to experience pain.
Screening and Assessment	Teasing apart pain and ED symptoms is challenging.	(ANESTH_1) It’s hard to know… do they really meet those criteria? What’s the difference between that [a pain condition] and an eating disorder is not always clear. Is this a variant of ARFID or something separate? So we end up seeing a number of those kids where it’s not clear. Is it an overlapping condition? Is it two separate things or is all the same thing?	(EATING_3) It’s really challenging to tease apart always like what is the pain piece and what is secondary to nutrition or serving like some sort of psychosocial function? (GI_5) It [the presenting problem of the patient] was a combination of fear of pain and fear of weight gain, you know, and it was just… it was hard to parse out.
Screening and Assessment	Parent involvement	(GI_10) Parents need to be involved and bought in…they aren’t always receptive to the psych referral. Many parents are committed to a physical explanation for their child’s symptoms- be it pain or nausea or weight loss.	(PAIN_9) I’m providing active coaching, education, modeling, and sending parents home with a plan because they are the ones who are with their kids the most. It’s not me.
Intervention	Patients must be medically stable to participate in pain treatment.	(ADOLMED_2) They have to be strong enough nutritionally to do physical therapy, but they can’t restore nutritionally because of chronic pain and if they are engaging in physical activity.	(PAIN_4) you would stop everything and make feeding weight like weight restoration the sole purpose of treatment. So until there are signs, either until their weight restored or until their signs, their body is now like responding and is getting enough calories in to be able to function.
Intervention	Parents’ buy-in to diagnosis(es) impacts intervention.	(ADOLMED_7) I work closely with the parents, because if they aren’t on board with the diagnosis or treatment recommendations we aren’t going to get very far. (ANESTH_9) I work hard to get parents on board. It’s interesting, functional pain can be challenging to get buy in from parents, but it’s been even harder for me to get parents to believe that their child might also have an eating disorder.	(EATING_8) It’s tough. I had a teen who was struggling with persistent abdominal pain and nausea. She was definitely restricting food to avoid these things. Parents did not want to stop taking her for medical tests, even though our team knew that this was a functional problem related to an eating disorder. They didn’t understand how therapy could help.
Intervention	Family Based Treatment	(ADOLMED_3) FBT is the gold standard as you know for eating disorder treatment. I still think it’s relevant for these kids who also have pain issues. These kids need to be eating and having parents take control is the best way to ensure that. If we leave it up to the eating disorder or pain, the kid isn’t going to eat.	(EATING_5) I’ve had like a few cases where we kind of had to use a family- based treatment approach to kind of encourage parents to help their kids push on and eat while trying to manage some of the pain. (PAIN_2) The FBT model would map on well to what we do for pain treatment. We do a lot of externalizing the pain. Parents are very involved in treatment and we train parents to be validating that they’re asking their kid to push through pain… like going to school, engaging in physical activity, or for these kids, eating. There’s no negotiation.
Intervention	ACT and CBT combination	(ANESTH_5) I know our psychologists use CBT for pain and some ACT strategies for pain. I’m sure they could also be helpful for kids who aren’t eating or are afraid of certain foods… gaining weight… I think that might be helpful, but that’s not my area of expertise.	(PAIN_4) We need to provide some kind of coping skills to navigate in the world. So I would use some ACT approaches infused with CBT. (EATING_6) It could actually work well to do some combination of interventions for pain and eating… I’m thinking possibly ACT and CBT. For eating it is especially important to have parents play a significant role, though.
Intervention	Exposure therapy	(ADOLMED_7) I recently had a patient 11 years old, and she experienced mild symptoms that became more severe, and she completely refused to eat. She agreed with the NG tube placement was discharged with a tube. So she received NG tube treatment and went to exposure therapy and after starting exposure therapy, she started to eat more and more, and then we removed the NG tube, which was great.	(EATING_8) I think oftentimes if, for example, the patient presents more like ARFID, the treatment is often exposure like we need to get you eating a wider variety of foods. We need to get you eating more food or like a higher volume. And I think that’s oftentimes like a lot of times with pain, like, for example, like fear of movement, like exposure, like you have to get out and do it as much as you don’t want to do it in your brain, you have to do it.
Intervention	Tube feeding	(ADOLMED_2) What I have found is that it doesn’t seem physiologically to help pain, whether they have a G tube like an NG tube or an NJ tube, pain is pain. (ANESTH_1) We turn the pump away or we put a bag over it again, just the visual cues, right? And some of the kids will still know. So it becomes really hard to suss out, you know, is this an eating disorder? Or, you know, is it a physiologic change in the gut that just makes it very painful to eat? And then, of course, they don’t eat because it hurts.	(PAIN_1) And when we first started, I feel like we had a slew of kids that came with feeding tubes. And I know that other intensive pain programs that know to the feeding tube like you have to be off the tube before you can do our program. And I think we learned that the hard way that there is actually a reason people are saying no to that because it really is a whole separate thing and not something that anyone on our team was equipped to manage.
Intervention	Medication	(ADOLMED_4) We often use cyproheptadine when patients have eating issues and are also restricting food for various reasons, sometimes including pain. (ADOLMED_7) Meds are something that we try not to use in these cases, but some things are helpful. SSRIs, cyproheptadine, um sometimes over the counter antacids can help. It depends. Most of the time medication isn’t the most helpful way to help these kids.	No comment
Intervention	Difficult to find setting appropriate for addressing both symptoms	(ADOLMED_4) Unfortunately, we don’t accept patients with chronic pain to our eating disorder program. It’s just part of our criteria. We are able to address mild pain complaints, such as discomfort due to fullness, nausea or constipation… if they have pain lasting for more than three months, we can’t take them.	(PAIN_2) I work in an interdisciplinary pain treatment program, and we have had several patients now that have come into our program. The program takes kids that have the highest levels of pain and impairment, or they’re being evaluated for the program. One of our criteria that we actually had to make because we were running into it so much was nutritional stability and no concerns for body image or weight loss, because we were seeing more and more patients come in with comorbid eating disorder and chronic pain symptoms.

**Table A2:** Theme 2: Clinician Training Themes, Codes, and Quotes.

Subtheme	Code	Physician Quotes	Psychologist Quotes
Training Experience	Clinicians feel undertrained in non-specialty.	(ANEST_1) Given that it was several ago, we probably received something that was reasonable for the time (eating disorder training). Given the greater awareness and so on now, would that be that amount then be enough now? Probably not. (ADOLMED_2) We got 0-2 h max in functional pain disorders and somatic symptoms. You know, it might be a didactic lecture or something, but it’s not a common thing that falls into adolescent medicine.	(PAIN_1) During graduate school and post grad, I didn’t get a lot of experience working with kids or teens with eating disorders. I don’t feel super confident in working with the population for that reason. (PAIN_10) I did get some training in pain management, both with adults and kids. Zero training in eating disorders. It was like never even a topic that was brought up. It was like they were separate worlds.
Training Experiences	Clinicians learn more in practice or on the job about comorbid EDs and pain than in graduate or medical school.	(ADOLMED_7) I have career long experience with eating disorders. In regards to pain, I don’t have any training. Of course, I have attended some workshops. I’ve talked with colleagues at the hospital. (ADOLMED_4) I got more experience with pain patients on the job than in med school trainings.	(EATING_8) I haven’t really had much pain training, unfortunately. I think it would have been a nice area to focus more on, but I’ve experienced it. I shadowed someone when I was on internship doing consultation liaison work and there was a case or two that was more related to pain. And I definitely see pain, chronic pain, even on the eating disorder inpatient unit. (EATING_8) You kind of learn as you experience those patients in clinic and have people to consult and supervise and help you with stuff, but nothing that was in my formal training. (PAIN_3) A lot of us are talking about how we haven’t trained much in eating disorders, and we’ve had to kind of learn on the job in the moment. And I think it’s kind of just because there’s a lack of integration into graduate or doctoral training.
Desired Training	Opportunities for exposure to population during post-doctoral training.	(ADOLMED_3) I would have loved to have more coursework on this in med school or I mean, coursework may not be possible, but just it’s so interesting. The intersection of these complex medical presentations with these psychiatric presentations is something that I think people don’t necessarily get a ton of exposure to until they are like physicians and on their own. (ANESTH_9) I saw some kids with eating disorders during my residency, but until I maybe got an hour or two lecture in med school on eating disorders. There wasn’t much.	(PAIN_7) There were some peds psych experiences that involved eating disorders on internship. It was a mix kind of child clinical slash pediatric psychology internship. There was no specific eating disorders program at the site.
Desired Training	Clinicians want more training in assessing functional/somatic symptoms and EDs.	(ADOLMED_7) It’s unfortunate, because the pain these kids have is real and we don’t always do a great job of validating functional stuff in the medical world. I’m not confident that I have the best language to respond when kids come in with functional pain or somatic symptoms. I would love more training on that.	(PAIN_10) I think I need more education on how to assess for eating disorders. I think we all need a lot more time to be able to assess especially if we aren’t super streamlined in assessing for eating disorders or pain.
Desired Training	Clinicians want training to improve identification of treatment targets.	(ADOLMED_2) It would be helpful to learn what I can do as a physician to better support the treatment targets of psychotherapy. I do work closely with the psychologists on our team, but I’m not well versed in the treatment targets for functional pain. I’d like to learn more. (ADOLMED_3) I think training in adolescent medicine could be improved for chronic functional pain. I think that since we don’t really know how to treat functional pain, we don’t know how to treat the malnutrition caused by functional pain. As medical physicians were not great at convincing parents what the illness is and how to push through functional pain… that although it seems mean to make your kid eat even though they have pain, you have to.	(EATING_3) It would be great for someone to develop a training with a panel of therapists to treat functional abdominal pain and functional nausea since we are sent so many patients with that chief complaint from the community. (PAIN_2) We just need to be doing a better job of identifying when an eating disorder is present and making appropriate treatment decisions and knowing enough to be able to know when to treat someone for an eating disorder versus when to treat pain.
Desired Training	Clinicians seeking training with a biopsychosocial lens.	(ANESTH_9) I think medical school creates too many subspecialists… they only travel down their isolated pathway in their tiny little subspecialty, and then when they hit a dead end, like, oh, I can’t figure out what’s wrong with you, gee, I wonder if it’s mental health? Then they [pain physicians] start referring them. We’d much rather see people up front. Like truly at the same moment, like, oh, you might have Crohn’s disease, but before we do a colonoscopy, let’s have you see our psychologist. In an ideal world.	(PAIN_10) I think it would be helpful if patients were to get to us sooner. It’s not the fault of any provider in particular… I just don’t think there’s enough training on somatic symptoms or functional pain, and how to go about that. Like, how the combination of biology and psychology, and environment can drive a lot of these things.

**Table A3:** Theme 3: Collaboration and Consultation Themes, Codes, and Quotes.

**Subtheme**	**Code(s)**	**Physician Quotes**	**Psychologist Quotes**
Referrals	Clinicians refer cases to other providers when they do not feel they have the expertise.	(ANESTH_1) I am not going to diagnose anybody with an eating disorder. I’m not qualified to do that, but I am qualified to be suspicious and make a referral.	(GI_10) If ever I felt like someone needed true treatment for an eating disorder, I’m going to refer because I don’t have the expertise for that. So, I don’t typically do the weight focus. I just say we have to stop the weight loss. How do we do that? Let’s come up with some strategies and go from there.
Care Setting	Communicating with providers in different care settings.	(ADOLMED_2) I collaborate with mental health people, whether they’re in the community or in our own program. I take the time to go and reach out to all of those people, I talk to community therapists, I call psychiatrists in the community…everyone needs to be talking to one another.	(EATING_6) Community psychologists and therapists sometimes reach out to us to see if we can provide any education on eating disorder treatment, especially when it’s a complex presentation- like a kid with pain and restrictive eating.
Care Setting	Clinicians see the benefit in working with adolescents with chronic pain and ED symptoms in interdisciplinary settings.	(ADOLMED_4) We are very much an interdisciplinary team. We have adolescent medicine physicians and fellows. The patients and then psychologists and social workers meet with patients for more like therapy side. We do have a dietitian who will sometimes meet with families and nursing staff, and that’s on the outpatient level. But if we’re on the inpatient unit, which is FBT informed, it’s, you know, the whole gamut. Again, nursing staff, PCAs, psychologists, physicians. So yeah, we very much collaborate on cases.	(GI_5) I really like that interdisciplinary approach. I think it helps patients move and improve a lot quicker than, you know, seeing me for outpatient and then seeing their doctor every three months. I just feel like it’s better when we’re all a team and presenting information in a uniform fashion.
Care Setting	It is challenging to find a higher level of care options for kids with both EDs and chronic pain.	(ADOLMED_7) In the inpatient intensive rehab program for kids who have been affected by chronic pain, they [patients] can’t walk, could be in a wheelchair, can’t attend school… they manage everything except for eating. They must be able to eat. Otherwise, they’re not able to participate, which makes no sense to me. They have to be strong enough nutritionally to do physical therapy, but they can’t restore nutritionally because of chronic pain. Our eating disorder program doesn’t accept kids with chronic pain either.	(PAIN_4) And our inpatient pain program really struggles, because part of their criteria... like they won’t take kids who have eating problems because they only can focus on the pain. They don’t think they have the capacity to do the additional things that come with eating disorder treatment. (EATING_6) We get really stuck… we are forced to decide what this kid needs more at the time, but often times neither options of higher level of care are able to address the intertwined issues.
Collaboration	Clinicians feel confident in knowing who to ask/consult within the interdisciplinary team.	(ANESTH_6) I know enough to know when I need to consult with someone on a case. We are an interdisciplinary team, so if there’s functional pain or psychological distress in the context of eating I know I need to get this patient to see psychology.	(GI_5) I work right with a gastroenterologist and we have nursing and dietitians and a speech language pathologist with us, so we’re getting the whole picture. I know I have support when things come up. (EATING_8) Sometimes there’s comorbid mental and physical health conditions, that’s why it’s good to have a whole team to look at the bigger picture. I can ask someone on my team to see the patient and give me insight.
Collaboration	Importance of defining roles in treatment team	(ADOLMED_2) If the patient’s primary problem appears to be phobia, fear related/choking type or food allergy fear of having an allergic reaction is fed by a body image-driven eating disorder then I collaborate with mental health people. I collaborate with dietitians, and I collaborate with GI. I take the time to go and reach out to all of those people, but if their primary problem is functional, I say that they should see a therapist.	(EATING_3) It’s so important to collaborate on our teams. I don’t know how many calories a patient should be consuming. I don’t have that expertise, so I loop in their dietitian if there are questions. I also don’t know if they’re orthostatic by heart rate or blood pressure unless they’re following with a pediatrician. We need to work together.
